# Associations between systemic inflammatory indices and the risk of renal function decline in patients with type 2 diabetes mellitus: a retrospective cohort study

**DOI:** 10.3389/fendo.2025.1538704

**Published:** 2025-08-29

**Authors:** Zhi Shang, Hai-Dong Zhang, Hui Qian, Yue-Ming Gao, Song-Tao Feng

**Affiliations:** ^1^ Department of Cardiology and Institute of Vascular Medicine, Peking University Third Hospital, Beijing, China; ^2^ Department of Nephrology, Peking University Third Hospital, Beijing, China; ^3^ Jiangsu Key Laboratory of Medical Science and Laboratory Medicine, Department of Laboratory Medicine, School of Medicine, Jiangsu University, Zhenjiang, Jiangsu, China; ^4^ Department of Nephrology, Jiangsu University Affiliated People’s Hospital, Zhenjiang, Jiangsu, China

**Keywords:** type 2 diabetes mellitus, renal function, systemic immune-inflammation index, systemic inflammation response index, pan-immune-inflammation value

## Abstract

**Aims:**

This study aimed to investigate the associations between three systemic inflammatory indices, including the systemic immune-inflammation index (SII), systemic inflammation response index (SIRI), and pan-immune-inflammation value (PIV), and the risk of renal function decline in patients with type 2 diabetes mellitus (T2DM).

**Methods:**

We consecutively enrolled 9,537 patients with T2DM hospitalized at Peking University Third Hospital. The systemic inflammatory indices were calculated from baseline blood routine indicators. Renal function decline was defined as an estimated glomerular filtration rate decreasing by ≥ 40% from baseline. All participants were categorized into tertiles according to the systemic inflammatory indices. Restricted cubic spline (RCS) curves, multivariable Cox proportional hazard regression models, and receiver operating characteristic (ROC) curves were used for analysis.

**Results:**

A total of 1,495 outcome events were recorded during the follow-up. The RCS analysis suggested a non-linear association of systemic inflammatory indices with the risk of renal function decline (*P* for nonlinear < 0.001). Using the lowest tertile as reference, multivariate Cox regression revealed that patients in the highest tertile of the three systemic inflammatory indices had a significantly higher risk of renal function decline (SII: HR=1.67, 95% CI=1.47–1.91, *P*<0.001; SIRI:HR=1.69, 95% CI=1.46–1.95, *P*<0.001; PIV: HR=1.58, 95% CI=1.38–1.81, *P*<0.001). The ROC curves showed that the SIRI was better than other two indices at predicting renal function decline.

**Conclusion:**

A significantly positive association was shown between systemic inflammatory indices and the risk of renal function decline in T2DM patients. Among these inflammatory indices, SIRI has relatively high predictive performance for renal function decline.

## 1 Introduction

Type 2 diabetes mellitus (T2DM), a global public concern, was estimated to affect 643 million people by 2030 (1). Chronic kidney disease (CKD) is a major microvascular complication of T2DM, affecting approximately 25–40% of all patients with T2DM (2). T2DM is also the leading cause of end-stage renal disease (ESRD) in the developed world (3). Chronic inflammatory response in T2DM was considered to be one of the factors responsible for the renal function decline (4). Overexpression of pattern recognition receptors with subsequent proinflammatory cytokines activation in innate immune cells, including lymphocytes, neutrophils and monocytes, might play a major role in deteriorating renal function in patients with T2DM (5–7). Previous studies found that a high neutrophil count was indicative of infection, a low lymphocyte count could indicate poor health and stress response, and monocytes could participate in the inflammatory response by undergoing differentiation (8). Platelets may also contribute to renal function decline by mediating inflammatory response (9). Systemic inflammatory indices considering different types of innate immune cells and/or platelets were believed to be able to reflect the intricate interplay between inflammation and immunity more effectively compared to assessing the number of these cells separately.

Considering the possible effect of chronic inflammation on renal function decline in T2DM, the positive association between neutrophil/lymphocyte ratio, monocyte/lymphocyte ratio and renal function decline, including risk of prevalent or new onset CKD, and needs of dialysis, was widely investigated by previous studies (10–13). Systemic inflammatory indices such as the systemic immune-inflammation index (SII), systemic inflammation response index (SIRI), and pan-immune-inflammation value (PIV), can reflect the status of systemic immune-inflammatory response (14, 15). The SII, a parameter calculated from platelet, neutrophil, and lymphocyte counts, was reported to be associated with the incidence of CKD (16), renal function decline (17), and progression to ESRD (18). Previous studies also found that higher levels of SII were associated with diabetic kidney disease (DKD) in patients with T2DM (19–21). The SIRI, calculated from neutrophil, monocyte, and lymphocyte counts, was also confirmed to be associated with CKD prevalence (22, 23) and renal function decline (17). The PIV, a newly defined inflammatory indice, was found to be associated with poor prognosis in patients with idiopathic membranous nephropathy (24), and was also demonstrated to be associated with the risk of postcontrast acute kidney injury (25).

Given that scant research focused on the relationship between the SII, SIRI, and PIV, and renal function decline in T2DM patients, we conducted this study to explore and elucidate the association of these inflammatory indices with renal function decline in T2DM patients, which might be implemented in early detection of renal function damage and improved management in T2DM patients.

## 2 Materials and methods

### 2.1 Study design and participants

**Figure 1:**
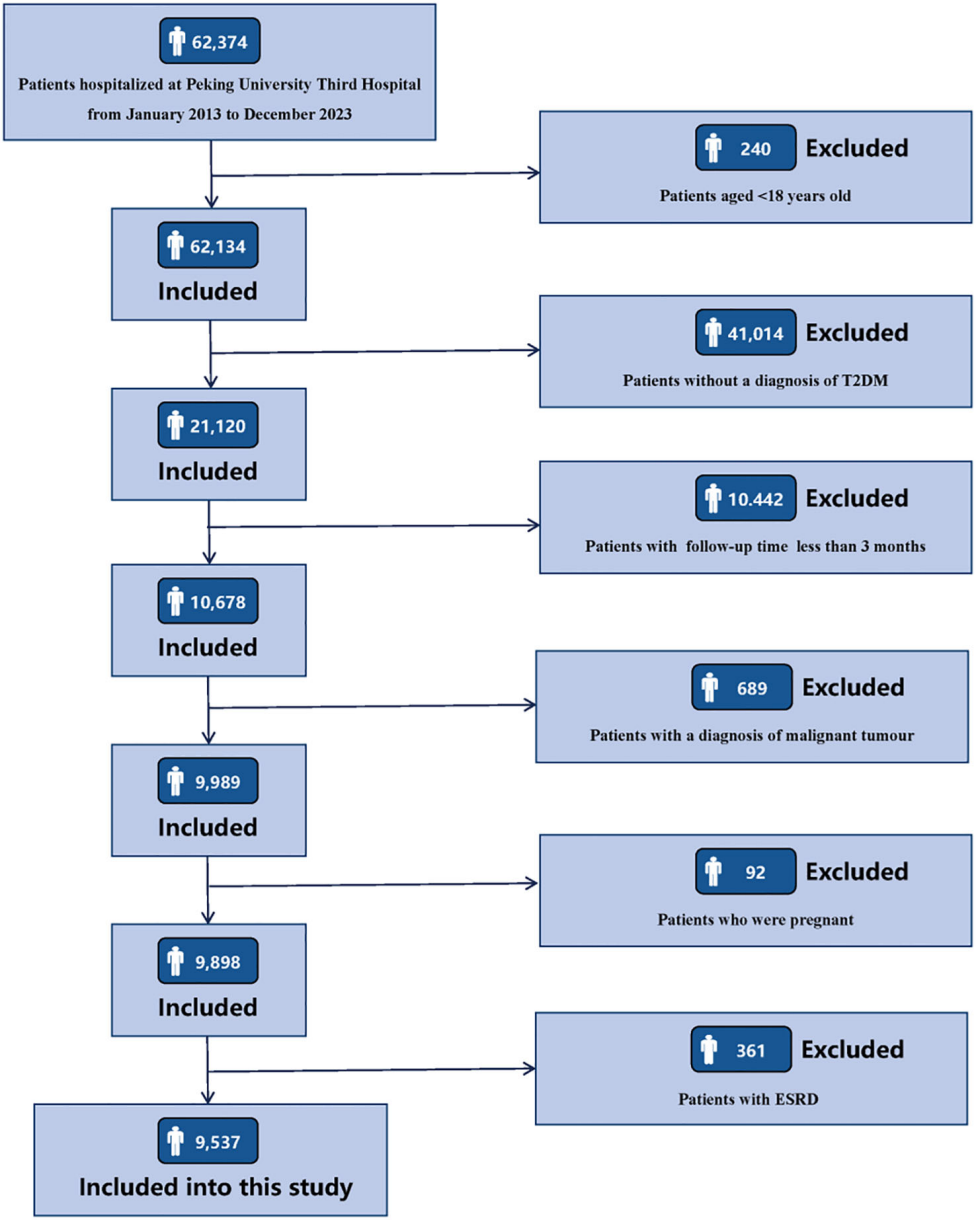
Flowchart of participants enrollment.

Clinical data of 62,347 consecutive patients admitted to Peking University Third Hospital from January 1, 2013 to December 31, 2023 were retrospectively collected. The inclusion criteria for the research subjects were as follows: (1) age ≥ 18 years old; (2) patients with a diagnosis of T2DM. The exclusion criteria for the research subjects are as follows: (1) patients with a follow-up period of less than 3 months; (2) patients complicated with malignant tumors; (3) patients who were pregnant; (4) patients with ESRD, including sustained estimated glomerular filtration rate (eGFR) < 15 mL/min/1.73 m^2^, maintenance hemodialysis, peritoneal dialysis, or kidney transplantation. Details of participants enrollment are shown in **Figure 1**. The research was conducted in line with the Declaration of Helsinki and received full approval from the Ethics Committee of Peking University Third Hospital (IRB00006761-M2024424). Appropriate consent was obtained from all participants.

### 2.2 Study outcomes

The study outcome was renal function decline, defined as a decrease of ≥ 40% in eGFR compared to baseline. The baseline eGFR was defined as the value of the patient tested during the first time hospitalization. Observation started at the time of first hospitalization, and the follow-up ended at the occurrence of the outcome events, or the time of the latest hospitalization, or outpatient visit. eGFR was calculated based on the Chronic Kidney Disease Epidemiology Collaboration equation (26).

### 2.3 Data collection and definitions

Baseline clinical characteristics, including age, sex, smoking status, body mass index (BMI), systolic blood pressure (SBP), diastolic blood pressure (DBP), the history of hypertension, hyperlipidemia, metabolic syndrome (MetS), coronary heart disease (CHD), heart failure (HF), and stroke, and the usage of insulin, renin-angiotensin-aldosterone system (RAAS) inhibitors, metformin, sodium-glucose cotransporter 2 (SGLT2) inhibitors, dipeptidyl peptidase 4 (DPP4) inhibitors, and glucagon-like peptide-1 receptor agonists (GLP1-RAs), were extracted from the electronic medical record system. Laboratory indicators, including white blood cell (WBC) counts, hemoglobin (HGB), neutrophil counts, lymphocyte counts, monocyte counts, platelet counts, serum creatinine (Scr), blood urea nitrogen (BUN), serum uric acid (SUA), eGFR, urine protein (0–4+), serum albumin (ALB), total cholesterol (TC), triglyceride (TG), high-density lipoprotein cholesterol (HDL-C), low-density lipoprotein cholesterol (LDL-C), fasting blood glucose (FBG), and glycosylated hemoglobin (HbA1c) were collected at the first time of hospitalization. The determination of blood routine indicators were measured by the Sysmex XE-2100 automated hematology analyzer. Blood biochemical indicators were measured by the HITACHI 7600 automatic biochemical analyzer.

Smoking status was divided into current, former, and nonsmokers. Current smokers were participants who had smoked regularly in the past six months. Former smokers were participants who have quit smoking for at least six months. Nonsmokers were participants who had never smoked throughout their lifetime (27). The BMI was calculated as body weight in kilograms divided by height in meters squared. Hypertension was defined as SBP ≥ 140 mmHg and/or DBP ≥ 90 mmHg, or a self-reported history of hypertension, or currently using antihypertensive drugs (28). Hyperlipidemia was defined as TC ≥ 6.22, or TG ≥ 2.26 mmol/L, or HDL-C < 1.04 mmol/L, or LDL-C ≥ 4.14 mmol/L, or a self-reported history of hyperlipidemia, or currently using lipid-lowering agents (29). The diagnosis of MetS required meeting 3 or more of the following criteria: (1) waist circumference ≥ 90 for men and ≥ 80 cm for women; (2) TG ≥ 150 mg/dL; (3) HDL-C < 40 mg/dL for men and < 50 mg/dL for women; (4) SBP ≥ 130 mmHg and/or DBP ≥ 80 mmHg and/or currently using antihypertensive agents; and (5) FBG ≥ 100 mg/dL (30).

### 2.4 Systemic inflammatory indices calculations

SII = platelet counts × neutrophil counts/lymphocyte counts (16).

SIRI = neutrophil counts × monocyte counts/lymphocyte counts (23).

PIV = neutrophil counts × platelet counts × monocyte counts/lymphocyte counts (15).

### 2.5 Statistical analysis

Continuous variables were presented as a mean ± standard deviation or a median (interquartile range), whereas categorical variables were presented as a frequency (percentage). Multiple imputations using chained equations (MICE) were used for missing value imputation (31). The Student’s t test or Mann Whitney U test was used to test group differences for continuous variables, and the Chi-square test was used for categorical variables. Restricted cubic spline (RCS) curves were used to depict the relationship between the inflammatory indices and the risk of renal function decline in patients with T2DM. All participants were categorized into tertiles based on the levels of SII, SIRI, and PIV. Cox proportional hazard regression was conducted to estimate the hazard ratios (HRs) and 95% confidence intervals (CIs). To adjust for potential covariates, three multivariate-adjusted models were developed as follows: Model 1, adjusted for sex and age; Model 2, adjusted for variables in Model 1 plus smoking status, hypertension, MetS, hyperlipidemia, the usage of insulin, the usage of RAASi, BMI, SBP, and DBP; Model 3 was adjusted for variables in Model 2 plus HbA1c, eGFR, urine protein, SUA, HGB, LDL-C, and HDL-C. Time to the event in each group of the systemic inflammatory indices (T1-T3) was presented by the Kaplan-Meier curve, and the log-rank test was used to test the statistical significance. Receiver operating characteristic (ROC) curves and the area under the curves (AUCs) were used to assess the predictive performance of the systemic inflammatory indices in predicting renal function decline. Furthermore, subgroup analyses were used to explore associations between patients with different characteristics, including age (≥ 60 or < 60 years), sex (male or female), BMI (≥ 28 or < 28 kg/m^2^), HbA1c (≥ 7.0 or < 7.0%), eGFR (≥ 60 or < 60 mL/min/1.73 m^2^), hypertension (yes or no), MetS (yes or no), HF (yes or no), and the usage of RAAS inhibitors (yes or no). All analyses were performed using R software (version 4.3.1). *P-*value < 0.05 (two-sided) was considered to be statistically significant.

## 3 Results

### 3.1 Baseline clinical characteristics of participants

This study included 9,537 T2DM patients with an average age of 62 ± 13 years, and 5,835 (61.18%) patients were male. During a median follow-up period of 26.10 (11.70, 56.23) months, a total of 1,495 participants experienced outcome events, with an incidence rate of 15.68%. Compared with patients without outcome events, patients who experienced outcome events were older, had higher levels of SBP, and had a higher prevalence of hypertension, MetS, CHD, HF, and stroke, had a higher proportion of usage of insulin, RAAS inhibitors, had higher levels of WBC counts, neutrophil counts, monocyte counts, Scr, BUN, SUA, urinary protein, TC, TG, FBG, and HbA1c, and had lower levels of DBP, HGB, lymphocyte, eGFR, serum ALB, and HDL-C, and had a higher proportion of usage of metformin, SGLT2 inhibitors, DPP4 inhibitors, and GLP1-RAs. It is worth noting that the levels of systemic inflammatory indices (SII, SIRI, PIV) in patients with outcome events were significantly higher than those in patients without outcome events (*P* < 0.001). The baseline clinical characteristics of the study population was shown in **Table 1**. The comparison of baseline characteristics of patients included in this study (n=9,537) and patients excluded due to a follow-up period less than 3 months (n=10,442) was shown in **Supplementary Table 1**.

**Table 1:** Baseline characteristics of patients with and without outcomes.

Variables	Overall (n = 9,537)	Patients with outcomes (n = 1,495)	Patients without outcomes (n = 8,042)	*P*-value
Age (years)	62 ± 13	66 ± 13	62 ± 13	<0.001
Male (n, %)	5,835 (61.18%)	890 (59.53%)	4,945 (61.49%)	0.162
Smoking status				0.192
Nonsmoker	5,443 (57.07%)	827 (55.32%)	4,616 (57.40%)	
Former smoker	3,368 (35.32%)	556 (37.19%)	2,812 (34.97%)	
Current smoker	726 (7.61%)	112 (7.49%)	614 (7.63%)	
BMI (kg/m^2^)	25.70 ± 8.17	25.54 ± 3.90	25.73 ± 8.74	0.178
SBP (mmHg)	136 ± 19	140 ± 21	136 ± 18	<0.001
DBP (mmHg)	78 ± 12	77 ± 13	78 ± 12	0.029
Hypertension (n, %)	6,663 (69.86%)	1,206 (80.67%)	5,457 (67.86%)	<0.001
Hyperlipidemia (n, %)	9,062 (95.02%)	1,427 (95.45%)	7,635 (94.94%)	0.440
MetS (n, %)	6,304 (66.10%)	1,080 (72.24%)	5,224 (64.96%)	<0.001
CHD (n, %)	4,465 (46.82%)	761 (50.90%)	3,704 (46.06%)	0.001
HF (n, %)	965 (10.12%)	307 (20.54%)	658 (8.18%)	<0.001
Stroke (n, %)	2,386 (25.02%)	425 (28.43%)	1,961 (24.38%)	0.001
Insulin (n, %)	3,167 (33.21%)	580 (38.80%)	2,587 (32.17%)	<0.001
RAAS inhibitors (n, %)	4,365 (45.77%)	853 (57.06%)	3,512 (43.67%)	<0.001
Metformin (n, %)	3,921 (41.11%)	416 (27.83%)	3,505 (43.58%)	<0.001
SGLT2 inhibitors (n, %)	776 (8.14%)	40 (2.68%)	736 (9.15%)	<0.001
DPP4 inhibitors (n, %)	676 (7.09%)	48 (3.21%)	628 (7.81%)	<0.001
GLP-1RAs (n, %)	246 (2.58%)	6 (0.40%)	240 (2.98%)	<0.001
WBC (×10^9^/L)	7.01 ± 2.31	7.35 ± 2.52	6.95 ± 2.27	<0.001
HGB (g/L)	135 ± 18	127 ± 21	137 ± 17	<0.001
Neutrophil (×10^9^/L)	4.44 ± 1.81	4.81 ± 1.93	4.37 ± 1.78	<0.001
Lymphocyte (×10^9^/L)	1.88 ± 0.64	1.76 ± 0.64	1.90 ± 0.64	<0.001
Monocyte (×10^9^/L)	0.44 ± 0.15	0.47 ± 0.16	0.44 ± 0.15	<0.001
Platelet (×10^9^/L)	211 ± 56	209 ± 59	212 ± 55	0.077
SII	457.01 (316.18, 687.9)	532.00 (352.97, 812.19)	446.04 (310.77, 663.70)	<0.001
SIRI	0.94 (0.62, 1.47)	1.17 (0.78, 1.84)	0.90 (0.60, 1.40)	<0.001
PIV	193.36 (121.33, 315.90)	236.09 (147.51, 387.58)	185.27 (117.77, 301.61)	<0.001
Scr (µmol/L)	78 (67, 92)	84 (70, 107)	78 (67, 90)	<0.001
BUN (mmol/L)	6.13 ± 2.49	7.32 ± 3.43	5.91 ± 2.20	<0.001
SUA (µmol/L)	344.94 ± 115.15	369.08 ± 108.64	340.45 ± 115.78	<0.001
eGFR (mL/min/1.73 m^2^)	82.75 ± 23.64	73.14 ± 27.68	84.54 ± 22.36	<0.001
Urine protein				<0.001
0-±	8,068 (84.60%)	984 (65.82%)	7,084 (88.09%)	
1+-2+	1,036 (10.86%)	281 (18.80%)	755 (9.39%)	
3+-4+	433 (4.54%)	230 (15.38%)	203 (2.52%)	
Serum ALB (g/L)	40.01 ± 4.53	37.67 ± 5.16	40.45 ± 4.26	<0.001
TC (mmol/L)	4.31 ± 1.24	4.45 ± 1.47	4.28 ± 1.19	<0.001
TG (mmol/L)	1.54 (1.10, 2.25)	1.60 (1.14, 2.45)	1.53 (1.09, 2.21)	<0.001
HDL-C (mmol/L)	1.01 ± 0.27	0.98 ± 0.27	1.01 ± 0.27	0.001
LDL-C (mmol/L)	2.58 ± 0.96	2.60 ± 1.04	2.57 ± 0.94	0.296
FBG (mmol/L)	8.03 ± 3.13	8.21 ± 3.54	7.99 ± 3.05	0.021
HbA1c (%)	8.07 ± 1.93	8.27 ± 2.09	8.03 ± 1.89	<0.001

BMI, body mass index; SBP, systolic blood pressure; DBP, diastolic blood pressure; MetS, metabolic syndrome; CHD, coronary heart disease; HF, heart failure; RAAS, renin-angiotensin-aldosterone; SGLT2, sodium-glucose cotransporter 2; DPP4, dipeptidyl peptidase 4; GLP-1RA, glucagon-like peptide-1 receptor agonist; WBC, white blood cell; HGB, hemoglobin; SII, systemic immune-inflammation index; SIRI, systemic inflammation response index; PIV, pan-immune-inflammation value; Scr, serun creatinine; BUN, blood urea nitrogen; SUA, serun uric acid; eGFR, estimated glomerular filtration rate; ALB, albumin; TC, total cholesterol; TG, triglyceride; HDL-C, high-density lipoprotein cholesterol; LDL-C, low-density lipoprotein cholesterol; FBG, fasting blood glucose; HbA1c, glycosylated hemoglobin.

### 3.2 Association between the inflammatory indices and the risk of renal function decline

**Figure 2:**
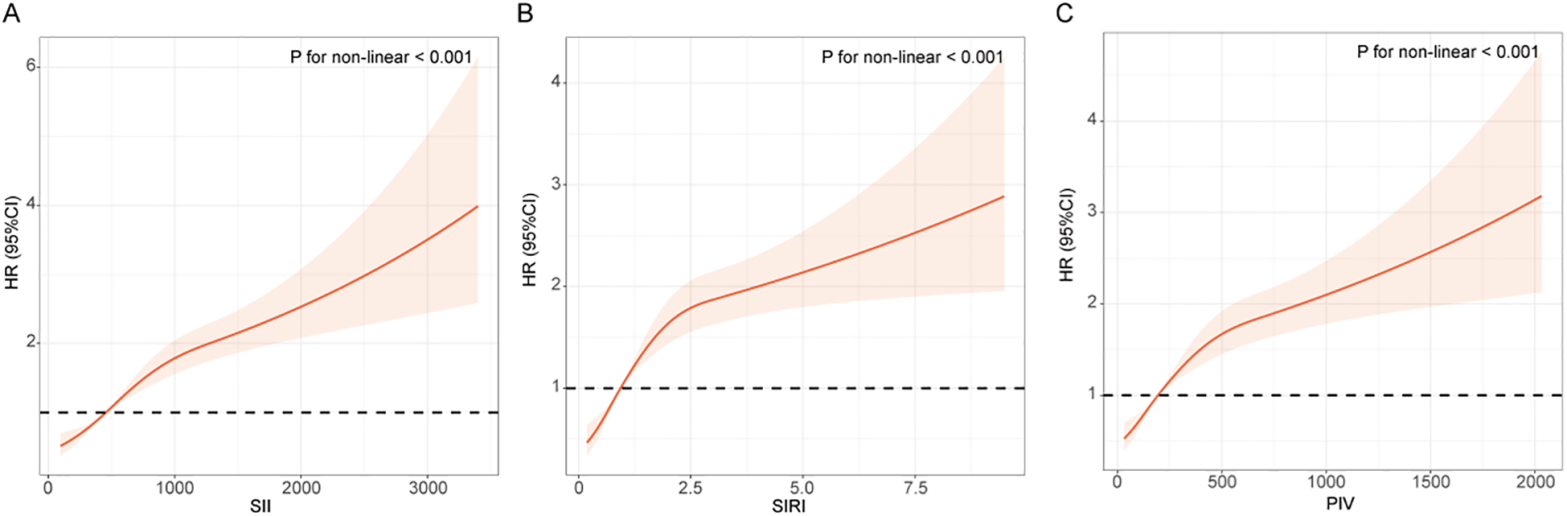
RCS curves between the inflammatory indices and the risk of renal function progression in patients with T2DM. **(A)** SII; **(B)** SIRI; **(C)** PIV. The RCS curve red solid line represented unadjusted HRs of the inflammatory indices across the whole range. The black dot line represents the reference line when HR = 1.

RCS curves were used to depict the dose-response relationship between the inflammatory indices and the risk of renal function decline in T2DM patients. **Figure 2** showed that there is a significantly positive association between the three inflammatory indices (SII, SIRI, PIV) and the risk of renal function decline (*P* for non-linear < 0.001).

As shown in **Table 2**, when these systemic inflammatory indices (including SII, SIRI, and PIV) were treated as continuous variables, each one-unit increment of them was significantly associated with an increased risk of renal function decline in the unadjusted model and the adjusted models, in which patients with increment of SIRI might had the highest risk of renal function decline compared to those with increment of SII and PIV (HR = 1.066, 95% CI: 1.030-1.104, *P* < 0.001 in the Model 3).

**Table 2:** Cox regression models for the association of systemic inflammatory indices with the risk of renal function progression.

	SII			SIRI			PIV		
	HR	95%CI	*P*-value	HR	95%CI	*P*-value	HR	95%CI	*P*-value
Unadjusted model									
Per unit increase	1.001	1.001-1.001	<0.001	1.233	1.196-1.271	<0.001	1.001	1.001-1.001	<0.001
Tertile 1	Ref			Ref			Ref		
Tertile 2	1.51	1.32-1.73	<0.001	1.54	1.34-1.78	<0.001	1.49	1.29-1.71	<0.001
Tertile 3	2.44	2.15-2.77	<0.001	2.44	2.13-2.79	<0.001	2.24	1.97-2.55	<0.001
*P* for trend			<0.001			<0.001			<0.001
Model 1									
Per unit increase	1.001	1.001-1.001	<0.001	1.209	1.170-1.249	<0.001	1.001	1.001-1.001	<0.001
Tertile 1	Ref			Ref			Ref		
Tertile 2	1.48	1.29-1.70	<0.001	1.46	1.27-1.69	<0.001	1.45	1.26-1.67	<0.001
Tertile 3	2.33	2.05-2.64	<0.001	2.22	1.93-2.55	<0.001	2.16	1.90-2.46	<0.001
*P* for trend			<0.001			<0.001			<0.001
Model 2									
Per unit increase	1.001	1.000-1.001	<0.001	1.187	1.149-1.226	<0.001	1.001	1.001-1.001	<0.001
Tertile 1	Ref			Ref			Ref		
Tertile 2	1.43	1.24- 1.64	<0.001	1.42	1.23-1.64	<0.001	1.41	1.23-1.62	<0.001
Tertile 3	2.25	1.98-2.56	<0.001	2.10	1.83-2.41	<0.001	2.05	1.79-2.34	<0.001
*P* for trend			<0.001			<0.001			<0.001
Model 3									
Per unit increase	1.000	1.000-1.000	<0.001	1.068	1.029-1.108	<0.001	1.000	1.000-1.001	<0.001
Tertile 1	Ref			Ref			Ref		
Tertile 2	1.32	1.15-1.51	<0.001	1.38	1.19-1.59	<0.001	1.31	1.14-1.50	<0.001
Tertile 3	1.67	1.47-1.91	<0.001	1.69	1.46-1.95	<0.001	1.58	1.38-1.81	<0.001
*P* for trend			<0.001			<0.001			<0.001

Model 1 was adjusted for sex and age; Model 2 was adjusted for variables in model 1 plus smoking status, hypertension, MetS, hyperlipidemia, the usage of insulin, the usage of RAAS inhibitors, BMI, SBP, and DBP; Model 3 was adjusted for variables in model 2 plus HbA1c, eGFR, urine protein, SUA, HGB, LDL-C, and HDL-C. SII, systemic immune-inflammation index; SIRI, systemic inflammation response index; PIV, pan-immune-inflammation value; MetS, metabolic syndrome; RAAS, renin-angiotensin-aldosterone; BMI, body mass index; SBP, systolic blood pressure; DBP, diastolic blood pressure; HbA1c, glycosylated hemoglobin; eGFR, estimated glomerular filtration rate; SUA, serun uric acid; HGB, hemoglobin; LDL-C, low-density lipoprotein cholesterol; HDL-C, high-density lipoprotein cholesterol.

**Figure 3:**
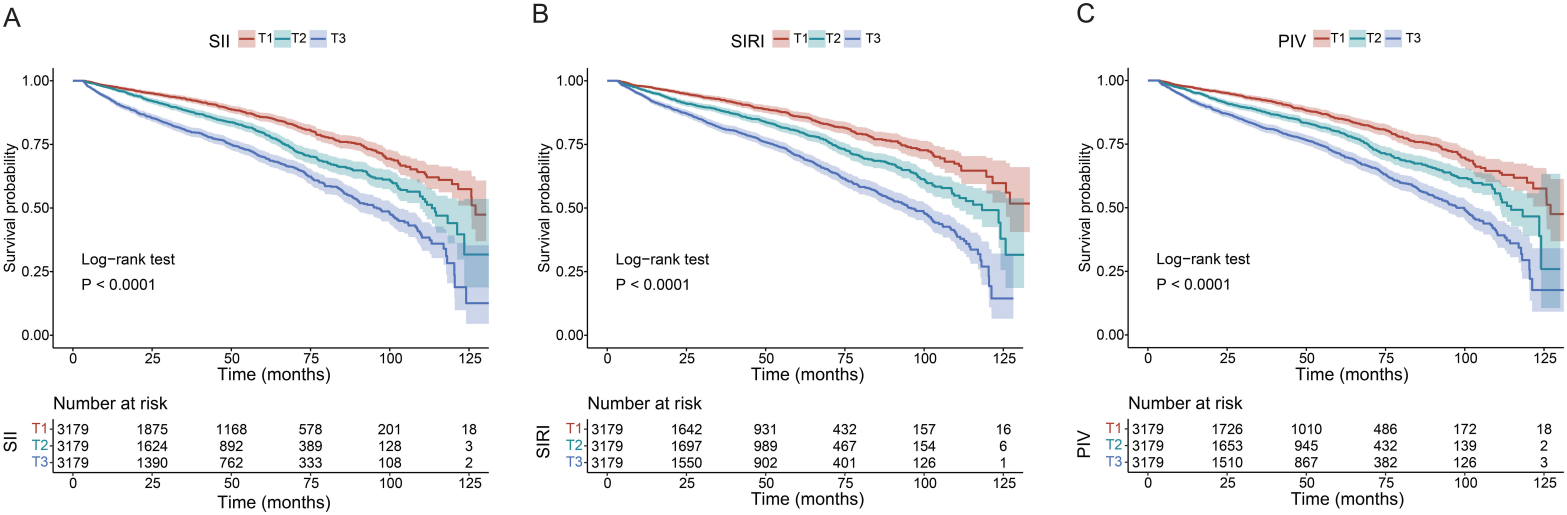
Kaplan-Meier curves of time to incident renal function progression. **(A)** SII; **(B)** SIRI; **(C)** PIV.

Participants were further categorized into tertiles based on their SII (T1 < 81.66, T2 358.60–589.00, T3 > 589.00), SIRI (T1 < 0.11, T2 0.72–1.24, T3 > 1.24), and PIV (T1 < 23.00, T2 142.30–262.20, T3 > 262.20). According to all three adjusted models, the highest SII tertile was linked to an increased risk of renal function decline (Model 1: HR 2.33, 95% CI 2.05–2.64, *P*<0.001; Model 2: HR 2.25, 95% CI 1.98–2.56, *P*<0.001; Model 3: HR 1.67, 95% CI 1.47–1.91, *P*<0.001). Using the lowest SIRI tertile as the reference, participants in the highest SIRI tertile had a significantly higher risk of renal function decline in both the unadjusted model (HR = 2.44, 95% CI: 2.13-2.79, *P* < 0.001) and the adjusted models (Model 1: HR 2.22, 95% CI 1.93–2.55, *P*<0.001; Model 2: HR 2.10, 95% CI 1.83–2.41, *P*<0.001; Model 3: HR 1.69, 95% CI 1.46–1.95, *P*<0.001). Using the lowest PIV tertile as the reference, participants in the highest PIV tertile had a significantly higher risk of renal function decline both in the unadjusted model (HR = 2.24, 95% CI: 1.97-2.55, *P* < 0.001) and the adjusted models (Model 1: HR 2.16, 95% CI 1.90–2.46, *P*<0.001; Model 2: HR 2.05, 95% CI 1.79–2.34, *P*<0.001; Model 3: HR 1.58, 95% CI 1.38–1.81, *P*<0.001). In addition, patients in the higher tertile of SII, SIRI, and PIV had a greater risk of renal function decline compared to patients in the lower tertile (all *P* for trend < 0.001). The Kaplan-Meier curves (**Figure 3**) were also performed to show that participants in the highest tertile of SII, SIRI, or PIV had a significantly higher risk of renal function decline than those in the lowest tertile (log-rank test, *P* < 0.001).

### 3.3 Predictive capability of systemic inflammatory indices in renal function decline

**Figure 4:**
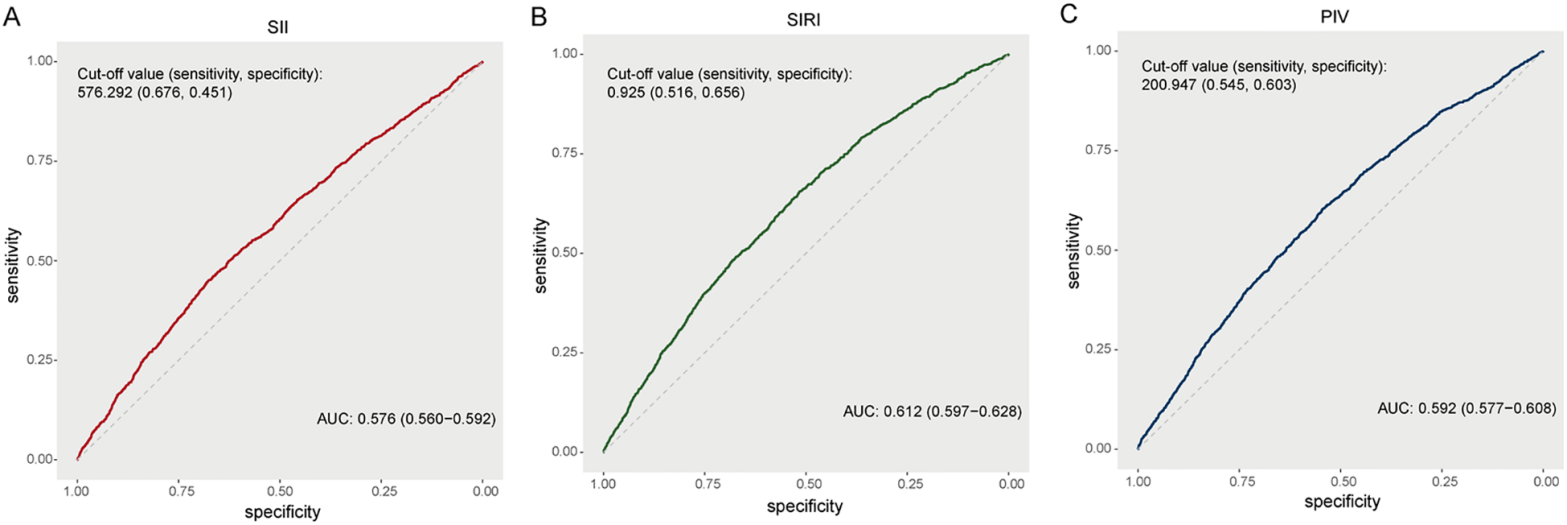
ROC curves showing the predictive performance of the SII **(A)**, SIRI **(B)**, and PIV **(C)**.

According to the ROC curves (**Figure 4**), the AUC of the SII for predicting renal function decline was 0.576 (95% CI: 0.560-0.592) with an optimal cut off value of 576.292 (sensitivity 67.6%, specificity 45.1%). The AUC of SIRI was 0.612 (95% CI: 0.597-0.628) with an optimal cut off value of 0.925 (sensitivity 51.6%, specificity 65.6%). The AUC of PIV was 0.592 (95% CI: 0.577-0.608) with an optimal cut off value of 200.947 (sensitivity 54.5%, specificity 60.3%). The ROC curves showed that the SIRI was better than other two inflammatory indices in predicting renal function decline.

### 3.4 Subgroup analysis

To verify the robustness of the positive association between the systemic inflammatory indices and the risk of renal function decline, subgroup analyses were conducted in accordance with the following stratification variables: age (≥ 60 or < 60 years), sex (male or female), BMI (≥ 28 or < 28 kg/m^2^), HbA1c (≥ 7.0 or < 7.0%), eGFR (≥ 60 or < 60 mL/min/1.73 m^2^), hypertension (yes or no), MetS (yes or no), HF (yes or no), and the usage of RAAS inhibitors (yes or no). As shown in **Supplementary Tables 2–4**, the subgroup analyses showed that usage of RAAS inhibitors would decrease the predictive capability of systemic inflammatory indices in the risk of renal function decline, and these systemic inflammatory indices showed a stronger association with renal function decline in participants without the usage of RAAS inhibitors. In addition, these systemic inflammatory indices had a robust positive correlation with the risk of renal function decline in all other subgroups.

## 4 Discussion

We recorded 1,495 outcome events in the 9,537 included T2DM patients during a median follow-up of 26.1 months. Patients with outcome events had a worse general condition in blood tests, higher levels of systemic inflammatory indices and higher proportion of usage of RAAS inhibitors compared to those without the outcomes. A non-linear association of the SII, SIRI, and PIV with the risk of renal function decline was detected. Patients in the highest tertile of the three systemic inflammatory indices had a significantly higher risk of renal function decline compared to the patients in the lowest tertile. Among these three indices, SIRI suggested a highest risk of renal function decline and was better at predicting renal function decline than the other two indices. Subgroup analysis suggested a robust positive correlation of these systemic inflammatory indices with the risk of renal function decline in all subgroups except that usage of RAAS inhibitors would significantly lower the associated risk of renal function decline with the inflammatory indices.

While previous studies have well-established the positive association of systemic inflammatory indices with mortality and cardiovascular events in T2DM patients (4, 32–34), suggesting the predictive capability of systemic inflammatory indices in survival of T2DM patients. There are also some studies focusing on the relationship between systemic inflammatory indices and kidney diseases. Studies have demonstrated that higher level of SII was associated with higher prevalence and increased risk of renal function decline of DKD, one of the most common microvascular complications of diabetes mellitus (19, 35, 36). In addition, SIRI was also demonstrated to be an independent risk factor for DKD diagnosis, but the number of cases included in this study was relatively small (35). T2DM could not only induce DKD and deteriorate eGFR decline in DKD, but could also enhance renal function decline in CKD through multiple processes, in which inducing chronic inflammatory response might be one of the factors responsible (4). Higher levels of SII and SIRI have been found to be associated with greater prevalence of CKD (4, 22, 23, 37), SII was useful in predicting the progression of CKD into ESRD in pediatric patients (38), and SIRI was also revealed to independently predict CKD progression and was associated with advanced CKD in CKD patients (8). These results indicated that systemic inflammatory indices might be able to predict the renal function decline in T2DM patients. Our study remained to be the first investigation studying the association between systemic inflammatory indices and renal function decline in T2DM patients, and the predictive capability of these indices in renal function decline in T2DM patients.

Our study demonstrated that SIRI was better at predicting renal function decline than the other two indices, suggesting that increased count of neutrophils and especially monocytes rather than platelet might play a more important role in the T2DM associated renal function decline. Neutrophils activation in CKD could worsen kidney injury and contribute to the development of kidney fibrosis through release of reactive oxygen species and neutrophil elastase (39), while monocytes infiltrate into kidney tissue with enhanced transendothelial migration capacity (40, 41), differentiate into macrophages and deteriorate renal injury by exceeding the number of other immune cells in CKD (42). Though research data has shown that macrophage was responsible for the adipose tissue inflammation, impaired insulin production and initiation of chronic renal inflammation in T2DM (5), the specific mechanism of monocyte/macrophage and neutrophil in inducing T2DM associated renal function decline remains to be further investigated. It should be noted that we only stated the association between the increased inflammatory indices and renal function decline in T2DM patients, the causality between increase in neutrophils and monocytes/macrophage and renal function decline in T2DM and its detailed mechanism should be based on basic research.

Though the ROC curve demonstrated that SIRI was better than other two inflammatory indices in predicting renal function decline in T2DM patients, the AUC was relatively low (0.612, 95% CI: 0.597-0.628), and the sensitivity and specificity (sensitivity 51.6%, specificity 65.6%) of the prediction model based on single SIRI was not qualified enough to predict the renal function decline accurately. Prediction models considering not only SIRI but also other factors involved in the previously published data including cystatin-C, serum ALB, HGB, 24-hour urinary protein, albuminuria, Scr, baseline eGFR, serum transferrin, SUA, serum bilirubin, blood pressure and drug administration (43–47) would increase the sensitivity and specificity of prediction model in predicting renal function decline in T2DM patients. A prediction model for accurately prediction of renal function decline would improve management in T2DM patients. Subgroup analysis suggested that usage of RAAS inhibitors would significantly lower the inflammatory indices associated risk of renal function decline, a reasonable explanation is that RAAS inhibitor might block the profibrotic effect of angiotensin II and aldosterone induced by neutrophil/monocyte activation (38).

Limitations of this study should be noted. Firstly, inherent data biases might be present as this was a retrospective single-centered study. Prospective multi-centered studies are needed for validation of the association of inflammatory indices with renal function decline in T2DM patients. Secondly, the participants collected in this study were hospitalized patients with T2DM, which may cause deficiencies in representativeness and universality. Thirdly, this study only confirmed the correlation between inflammatory indices and renal function decline, further investigation is required to comprehensively understand the underlying mechanisms revealed in this study. Fourthly, there are some difference between the baseline characteristic of patients included in this study and patients excluded from this study due to short follow-up period, which may lead to potential selection bias. Finally, we could not distinguish whether the indices associated renal function decline in T2DM patients led to DKD or non-DKD CKD as the raw pathological data was unavailable.

## 5 Conclusion

A significant and positive association was shown between the elevated SII, SIRI, and PIV, and the risk of renal function decline in T2DM patients. Among these inflammatory indices, SIRI has relatively high predictive performance for renal function decline.

## Data Availability

The original contributions presented in the study are included in the article/**Supplementary Material**. Further inquiries can be directed to the corresponding authors.

## References

[1] Sun H Saeedi P Karuranga S Pinkepank M Ogurtsova K Duncan BB. IDF Diabetes Atlas: Global, regional and country-level diabetes prevalence estimates for 2021 and projections for 2045. Diabetes Res Clin Pract. (2022) 183:109119. doi: 10.1016/j.diabres.2021.109119, PMID: 34879977 PMC11057359

[2] Pugliese G Penno G Natali A Barutta F Di Paolo S Reboldi G. Diabetic kidney disease: new clinical and therapeutic issues. Joint position statement of the Italian diabetes society and the Italian society of nephrology on “The natural history of diabetic kidney disease and treatment of hyperglycemia in patients with type 2 diabetes and impaired renal function. J Nephrol. (2020) 33:9–35. doi: 10.1007/s40620-019-00650-x, PMID: 31576500 PMC7007429

[3] Bakris GL. Update on reducing the development of diabetic kidney disease and cardiovascular death in diabetes. Kidney Int Suppl. (2011) 2018) 8:1. doi: 10.1016/j.kisu.2017.10.002, PMID: 30675432 PMC6336218

[4] Yang C Yang Q Xie Z Peng X Liu H Xie C. Association of systemic immune-inflammation-index with all-cause and cause-specific mortality among type 2 diabetes: a cohort study base on population. Endocrine. (2024) 84:399–411. doi: 10.1007/s12020-023-03587-1, PMID: 38048013 PMC11076376

[5] Rohm TV Meier DT Olefsky JM Donath MY. Inflammation in obesity, diabetes, and related disorders. Immunity. (2022) 55:31–55. doi: 10.1016/j.immuni.2021.12.013, PMID: 35021057 PMC8773457

[6] Donath MY Dinarello CA Mandrup-Poulsen T. Targeting innate immune mediators in type 1 and type 2 diabetes. Nat Rev Immunol. (2019) 19:734–46. doi: 10.1038/s41577-019-0213-9, PMID: 31501536

[7] Meyerovich K Fukaya M Terra LF Ortis F Eizirik DL Cardozo AK. The non-canonical NF-κB pathway is induced by cytokines in pancreatic beta cells and contributes to cell death and proinflammatory responses *in vitro* . Diabetologia. (2016) 59:512–21. doi: 10.1007/s00125-015-3817-z, PMID: 26634571

[8] Tang L Deng Y Lai J Guo X Liu P Li S. Predictive effect of system inflammation response index for progression of chronic kidney disease in non-dialyzing patient. J Inflammation Res. (2023) 16:5273–85. doi: 10.2147/JIR.S432699, PMID: 38026247 PMC10659112

[9] Gomchok D Ge RL Wuren T. Platelets in renal disease. Int J Mol Sci. (2023) 24:14724. doi: 10.3390/ijms241914724, PMID: 37834171 PMC10572297

[10] Kuo YT Wang YY Lin SY Chang WD. Age and sex differences in the relationship between neutrophil-to-lymphocyte ratio and chronic kidney disease among an adult population in Taiwan. Clin Chim Acta. (2018) 486:98–103. doi: 10.1016/j.cca.2018.07.025, PMID: 30025754

[11] Zhang M Wang K Zheng H Zhao X Xie S Liu C. Monocyte lymphocyte ratio predicts the new-onset of chronic kidney disease: a cohort study. Clin Chim Acta. (2020) 503:181–9. doi: 10.1016/j.cca.2019.11.021, PMID: 31794768

[12] Kocak MZ Aktas G Duman TT Atak BM Kurtkulagi O Tekce H. Monocyte lymphocyte ratio as a predictor of diabetic kidney injury in type 2 diabetes mellitus; The MADKID Study. J Diabetes Metab Disord. (2020) 19:997–1002. doi: 10.1007/s40200-020-00595-0, PMID: 33553019 PMC7843868

[13] Yoshitomi R Nakayama M Sakoh T Fukui A Katafuchi E Seki M. High neutrophil/lymphocyte ratio is associated with poor renal outcomes in Japanese patients with chronic kidney disease. Ren Fail. (2019) 41:238–43. doi: 10.1080/0886022X.2019, PMID: 30942116 PMC6450582

[14] Islam MM Satici MO Eroglu SE. Unraveling the clinical significance and prognostic value of the neutrophil-to-lymphocyte ratio, platelet-to-lymphocyte ratio, systemic immune-inflammation index, systemic inflammation response index, and delta neutrophil index: an extensive literature review. Turk J Emerg Med. (2024) 24:8–19. doi: 10.4103/tjem.tjem_198_23, PMID: 38343523 PMC10852137

[15] Qi X Qiao B Song T Huang D Zhang H Liu Y. Clinical utility of the pan-immune-inflammation value in breast cancer patients. Front Oncol. (2023) 13:1223786. doi: 10.3389/fonc.2023.1223786, PMID: 37711203 PMC10499041

[16] Liu X Li X Chen Y Liu X Liu Y Wei H. Systemic immune-inflammation Index is associated with chronic kidney disease in the U.S. population: insights from NHANES 2007-2018. Front Immunol. (2024) 15:1331610. doi: 10.3389/fimmu.2024.1331610, PMID: 38449859 PMC10915063

[17] Wang S Zeng J Chen Y Sun F Ma H Zhang L. Association between systemic inflammation and worsening renal function in cardiovascular-kidney-metabolic syndrome. Am J Nephrol. (2025) 28:1–11. doi: 10.1159/000546130, PMID: 40294586

[18] Zhai Y Sun S Zhang W Tian H. The prognostic value of the systemic immune inflammation index in patients with IgA nephropathy. Ren Fail. (2024) 46:2381613. doi: 10.1080/0886022X.2024.2381613, PMID: 39039867 PMC11268256

[19] Guo W Song Y Sun Y Du H Cai Y You Q. Systemic immune-inflammation index is associated with diabetic kidney disease in Type 2 diabetes mellitus patients: evidence from NHANES 2011-2018. Front Endocrinol (Lausanne). (2022) 13:1071465. doi: 10.3389/fendo.2022.1071465, PMID: 36561561 PMC9763451

[20] Zhang X Fang Y Weng M Chen C Xu Y Wan J. Systemic immune-inflammation index as an independent risk factor for diabetic nephropathy: a retrospective, single-center study. PeerJ. (2024) 12:e18493. doi: 10.7717/peerj.18493, PMID: 39677959 PMC11639188

[21] Patro S Choudhary A Sharma V Mahajan A Sahoo D Pattnaik SS. Evaluating platelet-to-lymphocyte ratio and systemic immune-inflammation index as distinctive biomarkers in type 2 diabetes mellitus patients with and without proteinuria: a retrospective study. Cureus. (2025) 17:e79348. doi: 10.7759/cureus.79348, PMID: 40125168 PMC11929124

[22] Li X Cui L Xu H. Association between systemic inflammation response index and chronic kidney disease: a population-based study. Front Endocrinol (Lausanne). (2024) 15:1329256. doi: 10.3389/fendo.2024.1329256, PMID: 38455650 PMC10917959

[23] Huang P Mai Y Zhao J Yi Y Wen Y. Association of systemic immune-inflammation index and systemic inflammation response index with chronic kidney disease: observational study of 40,937 adults. Inflammation Res. (2024) 73:655–67. doi: 10.1007/s00011-024-01861-0, PMID: 38489048

[24] Kazan DE Kazan S. Systemic immune inflammation index and pan-immune inflammation value as prognostic markers in patients with idiopathic low and moderate risk membranous nephropathy. Eur Rev Med Pharmacol Sci. (2023) 27:642–8. doi: 10.26355/eurrev_202301_31065, PMID: 36734708

[25] Kurtul A Gok M. Preinterventional pan-immune-inflammation value as a tool to predict postcontrast acute kidney injury among acute coronary syndrome patients implanted drug-eluting stents: a retrospective observational study. Scand J Clin Lab Invest. (2024) 84:97–103. doi: 10.1080/00365513.2024.2330904, PMID: 38506475

[26] Levey AS Stevens LA Schmid CH Zhang YL Castro AF 3rd Feldman HI. A new equation to estimate glomerular filtration rate. Ann Intern Med. (2009) 150:604–12. doi: 10.7326/0003-4819-150-9-200905050-00006, PMID: 19414839 PMC2763564

[27] Barry SA Tammemagi MC Penek S Kassan EC Dorfman CS Riley TL. Predictors of adverse smoking outcomes in the Prostate, Lung, Colorectal and Ovarian Cancer Screening Trial. J Natl Cancer Inst. (2012) 104:1647–59. doi: 10.1093/jnci/djs398, PMID: 23104210 PMC3490843

[28] Williams B Mancia G Spiering W Agabiti Rosei E Azizi M Burnier M. 2018 ESC/ESH Guidelines for the management of arterial hypertension. Eur Heart J. (2018) 39:3021–104. doi: 10.1093/eurheartj/ehy339, PMID: 30165516

[29] Grundy SM Stone NJ Bailey AL Beam C Birtcher KK Blumenthal RS. 2018 AHA/ACC/AACVPR/AAPA/ABC/ACPM/ADA/AGS/APhA/ASPC/NLA/PCNA guideline on the management of blood cholesterol: A report of the american college of cardiology/American heart association task force on clinical practice guidelines. Circulation. (2019) 139:e1082–143. doi: 10.1161/CIR.0000000000000625, PMID: 30586774 PMC7403606

[30] Grundy SM Cleeman JI Daniels SR Donato KA Eckel RH Franklin BA. Diagnosis and management of the metabolic syndrome: an American Heart Association/National Heart, Lung, and Blood Institute Scientific Statement. Circulation. (2005) 112:2735–52. doi: 10.1161/CIRCULATIONAHA.105.169404, PMID: 16157765

[31] White IR Royston P Wood AM. Multiple imputation using chained equations: issues and guidance for practice. Stat Med. (2011) 30:377–99. doi: 10.1002/sim.4067, PMID: 21225900

[32] Zhang J Fan X Xu Y Wang K Xu T Han T. Association between inflammatory biomarkers and mortality in individuals with type 2 diabetes: NHANES 2005-2018. Diabetes Res Clin Pract. (2024) 209:111575. doi: 10.1016/j.diabres.2024.111575, PMID: 38346591

[33] Tuzimek A Dziedzic EA Beck J Kochman W. Correlations between acute coronary syndrome and novel inflammatory markers (systemic immune-inflammation index, systemic inflammation response index, and aggregate index of systemic inflammation) in patients with and without diabetes or prediabetes. J Inflammation Res. (2024) 17:2623–32. doi: 10.2147/JIR.S454117, PMID: 38707954 PMC11067916

[34] Urbanowicz T Michalak M Al-Imam A Olasińska-Wiśniewska A Rodzki M Witkowska A. The significance of systemic immune-inflammatory index for mortality prediction in diabetic patients treated with off-pump coronary artery bypass surgery. Diagnostics (Basel). (2022) 12:634. doi: 10.3390/diagnostics12030634, PMID: 35328187 PMC8947274

[35] Liu W Zheng S Du X. Association of systemic immune-inflammation index and systemic inflammation response index with diabetic kidney disease in patients with type 2 diabetes mellitus. Diabetes Metab Syndr Obes. (2024) 17:517–31. doi: 10.2147/DMSO.S447026, PMID: 38327734 PMC10849098

[36] Li J Wang X Jia W Wang K Wang W Diao W. Association of the systemic immuno-inflammation index, neutrophil-to-lymphocyte ratio, and platelet-to-lymphocyte ratio with diabetic microvascular complications. Front Endocrinol (Lausanne). (2024) 15:1367376. doi: 10.3389/fendo.2024.1367376, PMID: 38660516 PMC11039910

[37] Li L Chen K Wen C Ma X Huang L. Association between systemic immune-inflammation index and chronic kidney disease: A population-based study. PloS One. (2024) 19:e0292646. doi: 10.1371/journal.pone.0292646, PMID: 38329961 PMC10852278

[38] Kawalec A Stojanowski J Mazurkiewicz P Choma A Gaik M Pluta M. Systemic immune inflammation index as a key predictor of dialysis in pediatric chronic kidney disease with the use of random forest classifier. J Clin Med. (2023) 12:6911. doi: 10.3390/jcm12216911, PMID: 37959376 PMC10647735

[39] Bronze-da-Rocha E Santos-Silva A. Neutrophil elastase inhibitors and chronic kidney disease. Int J Biol Sci. (2018) 14:1343–60. doi: 10.7150/ijbs.26111, PMID: 30123081 PMC6097478

[40] Bernelot Moens SJ Verweij SL van der Valk FM van Capelleveen JC Kroon J Versloot M. Arterial and cellular inflammation in patients with CKD. J Am Soc Nephrol. (2017) 28:1278–85. doi: 10.1681/ASN.2016030317, PMID: 27799487 PMC5373444

[41] Cormican S Negi N Naicker SD Islam MN Fazekas B Power R. Chronic kidney disease is characterized by expansion of a distinct proinflammatory intermediate monocyte subtype and by increased monocyte adhesion to endothelial cells. J Am Soc Nephrol. (2023) 34:793–808. doi: 10.1681/ASN.0000000000000083, PMID: 36799882 PMC10125648

[42] Frąk W Kućmierz J Szlagor M Młynarska E Rysz J Franczyk B. New insights into molecular mechanisms of chronic kidney disease. Biomedicines. (2022) 10:2846. doi: 10.3390/biomedicines10112846, PMID: 36359366 PMC9687691

[43] Ahn KH Kim SS Kim WJ Kim JH Nam YJ Park SB. Low serum bilirubin level predicts the development of chronic kidney disease in patients with type 2 diabetes mellitus. Korean J Intern Med. (2017) 32:875–82. doi: 10.3904/kjim.2015.153, PMID: 28560862 PMC5583441

[44] Norris KC Smoyer KE Rolland C van der Vaart J Grubb EB. Albuminuria, serum creatinine, and estimated glomerular filtration rate as predictors of cardio-renal outcomes in patients with type 2 diabetes mellitus and kidney disease: a systematic literature review. BMC Nephrol. (2018) 19:36. doi: 10.1186/s12882-018-0821-9, PMID: 29426298 PMC5807748

[45] Russo GT Giandalia A Ceriello A Di Bartolo P Di Cianni G Fioretto P. A prediction model to assess the risk of egfr loss in patients with type 2 diabetes and preserved kidney function: The amd annals initiative. Diabetes Res Clin Pract. (2022) 192:110092. doi: 10.1016/j.diabres.2022.110092, PMID: 36167264

[46] Zhao L Zou Y Zhang J Zhang R Ren H Li L. Serum transferrin predicts end-stage renal disease in type 2 diabetes mellitus patients. Int J Med Sci. (2020) 17:2113–24. doi: 10.7150/ijms.46259, PMID: 32922172 PMC7484672

[47] Zou Y Zhao L Zhang J Wang Y Wu Y Ren H. Development and internal validation of machine learning algorithms for end-stage renal disease risk prediction model of people with type 2 diabetes mellitus and diabetic kidney disease. Ren Fail. (2022) 44:562–70. doi: 10.1080/0886022X.2022.2056053, PMID: 35373711 PMC8986220

