# Supplementary material for: Associations between systemic inflammatory indices and the risk of renal function decline in patients with type 2 diabetes mellitus: a retrospective cohort study
Source: Front Endocrinol (Lausanne). 2025 Aug 29;16:1538704. doi: 10.3389/fendo.2025.1538704 (PMC12425747; doi:10.3389/fendo.2025.1538704)
Supplement: Supplementary file 1 [file Table1.docx]

Supplementary Material

# Supplementary Tables

**Supplementary Table 1. The comparison of baseline chracteristics of patients included in this study (n=9,537) and patients excluded due to a follow-up period less than 3 months (n=10,442)**

| **Variables** | **Patients included in the study**  **(n = 9,537)** | **Patients excluded due to short follow-up time**  **(n = 10,442)** | ***P*-value** |
| --- | --- | --- | --- |
| Age (years) | 62±13 | 62±13 | 0.308 |
| Male (n, %) | 5,835 (61.18%) | 6,275 (60.09%) | 0.119 |
| Smoking status |  |  | 0.008 |
| Nonsmoker | 5,443 (57.07%) | 6,064 (58.1%) |  |
| Former smoker | 3,368 (35.32%) | 3,700 (35.4%) |  |
| Current smoker | 726 (7.61%) | 678 (6.50%) |  |
| BMI (kg/m^2^) | 25.70±8.17 | 25.57±6.47 | 0.220 |
| SBP (mmHg) | 136±19 | 137±20 | 0.002 |
| DBP (mmHg) | 78±12 | 79±12 | 0.007 |
| Hypertension (n, %) | 6,663 (69.86%) | 7,247 (69.40%) | 0.487 |
| Hyperlipidemia (n, %) | 9,062 (95.02%) | 9,806 (93.91%) | 0.001 |
| MetS (n, %) | 6,304 (66.10%) | 6,749 (64.63%) | 0.031 |
| CHD (n, %) | 4,465 (46.82%) | 4,508 (43.17%) | <0.001 |
| HF (n, %) | 965 (10.12%) | 1,276 (12.22%) | <0.001 |
| Stroke (n, %) | 2,386 (25.02%) | 2,647 (25.35%) | 0.601 |
| Insulin (n, %) | 3,167 (33.21%) | 3,217 (30.81%) | <0.001 |
| RAAS inhibitors (n, %) | 4,365 (45.77%) | 4,159 (39.83%) | <0.001 |
| WBC (×10^9^/L) | 7.01±2.31 | 7.03±2.56 | 0.601 |
| HGB (g/L) | 135±18 | 134±20 | <0.001 |
| Neutrophil (×10^9^/L) | 4.44±1.81 | 4.58±2.22 | 0.003 |
| Lymphocyte (×10^9^/L) | 1.88±0.64 | 1.82±0.72 | <0.001 |
| Monocyte (×10^9^/L) | 0.44±0.15 | 0.44±0.18 | <0.001 |
| Platelet (×10^9^/L) | 211±56 | 215±65 | 0.005 |
| Scr (µmol/L) | 78 (67, 92) | 78 (66, 93) | 0.069 |
| SUA (µmol/L) | 344.94±115.15 | 346.91±116.73 | 0.230 |
| eGFR (mL/min/1.73 m^2^) | 82.75±23.64 | 81.35±27.08 | <0.001 |
| Urine protein |  |  | <0.001 |
| 0-± | 8,068 (84.60%) | 8,644 (82.80%) |  |
| 1+-2+ | 1,036 (10.86%) | 1,408 (13.50%) |  |
| 3+-4+ | 433 (4.54%) | 390 (3.70%) |  |
| Serum ALB (g/L) | 40.01±4.53 | 39.92±4.95 | 0.152 |
| TC (mmol/L) | 4.31±1.24 | 4.27±1.26 | 0.023 |
| TG (mmol/L) | 1.54 (1.10, 2.25) | 1.49 (1.08, 2.14) | <0.001 |
| HDL-C (mmol/L) | 1.01±0.27 | 1.01±0.26 | 0.615 |
| LDL-C (mmol/L) | 2.58±0.96 | 2.52±0.95 | <0.001 |
| FBG (mmol/L) | 8.03±3.13 | 8.04±3.23 | 0.826 |
| HbA1c (%) | 8.07±1.93 | 7.88±1.87 | <0.001 |

**Supplementary Table 2. Subgroup analysis of the association between the SII and renal function progression**

| **Variables**  No./total | **Tertile 1**  **(81.66, 358.60)** | **Tertile 2**  **(358.60, 589.00)** | **Tertile 3**  **(589.00, 4082.10)** | ***P* for interaction** |
| --- | --- | --- | --- | --- |
| Age (years) |  |  |  | 0.377 |
| < 60 (3,697/9,537) | Ref | 1.623  (1.265, 2.082) | 2.668  (2.106, 3.379) |  |
| ≥ 60 (5,840/9,537) | Ref | 1.427  (1.213, 1.679) | 2.231  (1.920, 2.593) |  |
| Sex |  |  |  | 0.611 |
| Male (5,835/9,537) | Ref | 1.413  (1.145, 1.743) | 2.432  (1.998, 2.960) |  |
| Female (3,702/9,537) | Ref | 1.582  (1.323, 1.892) | 2.446  (2.071, 2.889) |  |
| BMI (kg/m^2^) |  |  |  | 0.394 |
| < 28 (7,360/9,537) | Ref | 1.493  (1.277, 1.744) | 2.509  (2.175, 2.894) |  |
| ≥ 28 (2,177/9,537) | Ref | 1.558  (1.176, 2.063) | 2.177  (1.652, 2.870) |  |
| HbA1c (%) |  |  |  | 0.082 |
| < 7.0 (3,280/9,537) | Ref | 1.861  (1.438, 2.407) | 2.895  (2.267, 3.698) |  |
| ≥ 7.0 (6,257/9,537) | Ref | 1.398  (1.189, 1.643) | 2.293  (1.975, 2.661) |  |
| eGFR (mL/min/1.73 m^2^) |  |  |  | 0.977 |
| < 60 (1,612/9,537) | Ref | 1.408  (1.079, 1.836) | 2.142  (1.675, 2.739) |  |
| ≥ 60 (7,925/9,537) | Ref | 1.419  (1.210, 1.665) | 2.216  (1.907, 2.575) |  |
| Hypertension |  |  |  | 0.919 |
| Yes (6,663/9,537) | Ref | 1.417  (1.214, 1.653) | 2.280  (1.974, 2.633) |  |
| No (2,874/9,537) | Ref | 1.506  (1.123, 2.019) | 2.356  (1.789, 3.104) |  |
| Mets |  |  |  | 0.790 |
| Yes (6,304/9,537) | Ref | 1.489  (1.267, 1.750) | 2.359  (2.026, 2.747) |  |
| No (3,233/9,537) | Ref | 1.449  (1.121, 1.873) | 2.514  (1.997, 3.164) |  |
| HF |  |  |  | 0.624 |
| Yes (965/9,537) | Ref | 1.252  (0.880, 1.779) | 1.827  (1.329, 2.510) |  |
| No (8,572/9,537) | Ref | 1.475  (1.272, 1.710) | 2.267  (1.970, 2.609) |  |
| RAAS inhibitors |  |  |  | **0.048** |
| Yes (4,365/9,537) | Ref | 1.292  (1.077, 1.551) | 2.063  (1.742, 2.443) |  |
| No (5,172/9,537) | Ref | 1.672  (1.363, 2.051) | 2.693  (2.222, 3.263) |  |

**Supplementary Table 3. Subgroup analysis of the association between the SIRI and renal function progression**

| **Variables**  No./total | **Tertile 1**  **(0.11, 0.72)** | **Tertile 2**  **(0.72, 1.24)** | **Tertile 3**  **(1.24, 14.28)** | ***P* for interaction** |
| --- | --- | --- | --- | --- |
| Age (years) |  |  |  | 0.175 |
| < 60 (3,697/9,537) | Ref | 1.803  (1.408, 2.311) | 2.533  (1.981, 3.240) |  |
| ≥ 60 (5,840/9,537) | Ref | 1.352  (1.135, 1.609) | 2.163  (1.841, 2.542) |  |
| Sex |  |  |  | 0.465 |
| Male (5,835/9,537) | Ref | 1.438  (1.177, 1.758) | 2.390  (1.984, 2.879) |  |
| Female (3,702/9,537) | Ref | 1.719  (1.401, 2.109) | 2.592  (2.119, 3.170) |  |
| BMI (kg/m^2^) |  |  |  | 0.570 |
| < 28 (7,360/9,537) | Ref | 1.608  (1.367, 1.891) | 2.511  (2.157, 2.925) |  |
| ≥ 28 (2,177/9,537) | Ref | 1.344  (0.998, 1.811) | 2.192  (1.650, 2.914) |  |
| HbA1c (%) |  |  |  | 0.142 |
| < 7.0 (3,280/9,537) | Ref | 1.702  (1.295, 2.237) | 2.951  (2.297, 3.791) |  |
| ≥ 7.0 (6,257/9,537) | Ref | 1.469  (1.243, 1.737) | 2.247  (1.915, 2.636) |  |
| eGFR (mL/min/1.73 m^2^) |  |  |  | 0.639 |
| < 60 (1,612/9,537) | Ref | 1.324  (0.9831, 1.783) | 1.867  (1.4225, 2.451) |  |
| ≥ 60 (7,925/9,537) | Ref | 1.487  (1.263, 1.751) | 2.165  (1.848, 2.535) |  |
| Hypertension |  |  |  | 0.202 |
| Yes (6,663/9,537) | Ref | 1.372  (1.167, 1.613) | 2.165  (1.861, 2.519) |  |
| No (2,874/9,537) | Ref | 1.857  (1.372, 2.512) | 2.670  (1.986, 3.590) |  |
| Mets |  |  |  | 0.193 |
| Yes (6,304/9,537) | Ref | 1.797  (1.381, 2.339) | 2.851  (2.224, 3.657) |  |
| No (3,233/9,537) | Ref | 1.797  (1.381, 2.339) | 2.851  (2.224, 3.657) |  |
| HF |  |  |  | 0.898 |
| Yes (965/9,537) | Ref | 1.454  (0.965, 2.190) | 2.002  (1.384, 2.894) |  |
| No (8,572/9,537) | Ref | 1.493  (1.282, 1.739) | 2.165  (1.868, 2.509) |  |
| RAAS inhibitors |  |  |  | **<0.001** |
| Yes (4,365/9,537) | Ref | 1.416  (1.170, 1.713) | 1.897  (1.582, 2.276) |  |
| No (5,172/9,537) | Ref | 1.565  (1.260, 1.944) | 2.937  (2.404, 3.588) |  |

**Supplementary Table 4. Subgroup analysis of the association between the PIV and renal function progression**

| **Variables**  No./total | **Tertile 1**  **(23.0, 142.3)** | **Tertile 2**  **(142.3, 262.2)** | **Tertile 3**  **(262.2, 2261.4)** | ***P* for interaction** |
| --- | --- | --- | --- | --- |
| Age (years) |  |  |  | 0.175 |
| < 60 (3,697/9,537) | Ref | 1.803  (1.408, 2.311) | 2.533  (1.981, 3.240) |  |
| ≥ 60 (5,840/9,537) | Ref | 1.352  (1.135, 1.609) | 2.163  (1.841, 2.542) |  |
| Sex |  |  |  | 0.192 |
| Male (5,835/9,537) | Ref | 1.504  (1.250, 1.810) | 2.110  (1.776, 2.508) |  |
| Female (3,702/9,537) | Ref | 1.463  (1.188, 1.802) | 2.507  (2.056, 3.058) |  |
| BMI (kg/m^2^) |  |  |  | 0.068 |
| < 28 (7,360/9,537) | Ref | 1.619  (1.384, 1.894) | 2.379  (2.051, 2.758) |  |
| ≥ 28 (2,177/9,537) | Ref | 1.103  (0.825, 1.474) | 1.813  (1.384, 2.375) |  |
| HbA1c (%) |  |  |  | 0.164 |
| < 7.0 (3,280/9,537) | Ref | 1.724  (1.335, 2.227) | 2.638  (2.077, 3.351) |  |
| ≥ 7.0 (6,257/9,537) | Ref | 1.379  (1.170, 1.625) | 2.078  (1.780, 2.426) |  |
| eGFR (mL/min/1.73 m^2^) |  |  |  | 0.481 |
| < 60 (1,612/9,537) | Ref | 1.283  (0.980, 1.681) | 1.739  (1.362, 2.222) |  |
| ≥ 60 (7,925/9,537) | Ref | 1.470  (1.251, 1.726) | 2.084  (1.785, 2.433) |  |
| Hypertension |  |  |  | 0.818 |
| Yes (6,663/9,537) | Ref | 1.400  (1.197, 1.638) | 2.062  (1.779, 2.391) |  |
| No (2,874/9,537) | Ref | 1.437  (1.068, 1.933) | 2.258  (1.709, 2.982) |  |
| Mets |  |  |  | 0.155 |
| Yes (6,304/9,537) | Ref | 1.401  (1.188, 1.651) | 2.023  (1.731, 2.364) |  |
| No (3,233/9,537) | Ref | 1.555  (1.203, 2.008) | 2.636  (2.083, 3.336) |  |
| HF |  |  |  | 0.632 |
| Yes (965/9,537) | Ref | 1.304  (0.911, 1.866) | 1.706  (1.247, 2.335) |  |
| No (8,572/9,537) | Ref | 1.486  (1.279, 1.726) | 2.077  (1.798, 2.400) |  |
| RAAS inhibitors |  |  |  | **0.024** |
| Yes (4,365/9,537) | Ref | 1.297  (1.078, 1.560) | 1.825  (1.534, 2.171) |  |
| No (5,172/9,537) | Ref | 1.595  (1.295, 1.965) | 2.568  (2.112, 3.123) |  |
